# Surgically resected lung adenocarcinoma: do heterogeneous GGNs and part-solid nodules on thin-section CT show different prognosis?

**DOI:** 10.1007/s11604-022-01345-3

**Published:** 2022-10-11

**Authors:** Hirofumi Koike, Kazuto Ashizawa, Shin Tsutsui, Minoru Fukuda, Shinji Okano, Keitaro Matsumoto, Takeshi Nagayasu, Sumihisa Honda, Masataka Uetani

**Affiliations:** 1 Departments of Radiology, Nagasaki University Graduate School of Biomedical Sciences, 1-7-1 Sakamoto, Nagasaki, 852-8501 Japan; 2 Clinical Oncology, Nagasaki University Graduate School of Biomedical Sciences, 1-7-1 Sakamoto, Nagasaki, 852-8501 Japan; 3 Clinical Oncology Center, Nagasaki University Hospital, 1-7-1 Sakamoto, Nagasaki, 852-8501 Japan; 4 Depatment of Pathology, Nagasaki University Hospital, 1-7-1 Sakamoto, Nagasaki, 852-8501 Japan; 5 Surgical Oncology, Nagasaki University Graduate School of Biomedical Sciences, 1-7-1 Sakamoto, Nagasaki, 852-8501 Japan; 6 Nagasaki University Graduate School of Biomedical Sciences, 1-7-1 Sakamoto, Nursing, Nagasaki, 852-8501 Japan

**Keywords:** Computed tomography, Pulmonary adenocarcinoma, Heterogeneous ground-glass nodule, Part-solid nodule

## Abstract

**Purpose:**

This study aimed to evaluate the clinical courses of patients with surgically resected stage IA pulmonary adenocarcinoma (Ad) who exhibited heterogeneous ground-glass nodules (GGNs) or part-solid nodules on thin-section computed tomography (TSCT) and to clarify the prognostic differences between them.

**Materials and methods:**

The cases of 242 patients with proven pulmonary Ad with heterogeneous GGN or part-solid nodule who underwent surgical resection were retrospectively reviewed. After surgery, they were examined pathologically. Disease-free survival (DFS) and overall survival (OS) were also investigated.

**Results:**

There were no cases of recurrent pulmonary Ad or death from the primary disease in the heterogeneous GGN group. In the part-solid nodule group, recurrent pulmonary Ad and death from the primary disease were observed in 12 and 6 of 181 patients, respectively. Heterogeneous GGNs were associated with significantly longer DFS than part-solid nodules (*p* = 0.042). While, there was no significant difference in OS between the two groups (*p* = 0.134). Pathological diagnoses were available for all 242 patients. 181 part-solid nodules were classified into 116 invasive Ads, 54 minimally invasive Ads (MIAs), and 11 Ad in situ (AIS) lesions, and 61 heterogeneous GGNs were classified into 18 invasive Ads, 25 MIAs, and 18 AIS lesions.

**Conclusion:**

Heterogeneous GGNs were significantly associated with longer DFS than part-solid nodules. Pathologically, there were significant differences between the heterogeneous GGNs and part-solid nodules.

## Introduction

Lung cancer is the leading cause of cancer-related mortality. In 2021, 12–13% of cancer patients were diagnosed with lung cancer for the first time, and 22% of all cancer deaths are due to lung cancer [1]. A previous study by the National Lung Screening Trial reported that low-dose computed tomography (CT) screening could reduce mortality associated with lung cancer in high-risk smokers [2]. The most common type of lung cancer is adenocarcinoma (Ad), which accounts for around 40% of all cases of lung cancer. Pulmonary Ad develops from small airway epithelial, type II alveolar cells, which secrete mucus and other substances [3, 4].

For pulmonary Ad patients who exhibit sub-solid nodules (SSNs) on thin-section CT (TSCT), which refers to both pure ground-glass nodules (GGNs) and part-solid nodules, accurate clinical staging is important for preoperatively determining the optimal extent of a surgical procedure [5]. Pure GGNs are focal nodular areas of hazy increased attenuation that do not obscure the underlying bronchial or vascular structures, whereas part-solid nodules are composed of a combination of ground-glass and solid components, which completely obscure the underlying lung parenchyma [6]. Part-solid nodules are considered to be more invasive than pure GGNs [3, 7–11]. According to a previous study, among part-solid nodules, heterogeneous GGNs, whose solid components can only be detected on TSCT with the lung window, increase in size slower than part-solid nodules, whose solid components can be detected on TSCT with both the lung and mediastinal windows [12]. However, the characteristics of heterogeneous GGNs remain unclear. Thus, the purpose of this study was to investigate the clinical courses of lung cancer patients with heterogeneous GGNs and to clarify the differences between heterogeneous GGNs and part-solid nodules.

## Methods

Institutional review board approval was obtained. The need for written informed consent was waived by the institutional review board because this study was retrospective.

### Patients

The cases of 321 patients who had undergone surgical resection for proven stage IA pulmonary Ad between 2006 and 2015 were retrospectively reviewed. As the number of patients with pure GGNs was small, and the prognosis of patients with solid nodules and pure GGNs was clarified in previous studies, patients with solid nodules and pure GGNs were excluded from the evaluation. Finally, this study included 242 consecutive patients (84 males, 158 females; age range: 39–93 years; mean age: 68.2 years) with proven stage IA pulmonary Ad (Table 1). All of these patients were identified in a retrospective review of medical records conducted at a single medical institution.

**Table 1:** Clinical characteristics of the patients with stage IA pulmonary Ad with heterogeneous GGN and part-solid nodules (*n* = 242)

Variables	Heterogeneous GGNs (*n* = 61)	Part-solid nodules (*n* = 181)	*P* value
Age	67.0 ± 8.9	68.6 ± 9.1	0.746
Sex (male: female ratio)	19/42 (45%)	65/116 (56%)	0.457
BI (number of patients, *n*)	231 (25)	246 (71)	0.406
Current smoker (*n*)	18	54	
Former smoker (*n*)	7	17	
Non-smoker (*n*)	36	110	
History of cancer	19	49	0.661
History of lung cancer surgery	0	5	0.428

Data are expressed as n or mean ± standard deviation (range) values. *Ad* adenocarcinoma, *GGN* ground-glass nodule, *BI* brinkman index (number of cigarettes smoked per day multiplied by number of years of smoking)

**Fig. 1:**
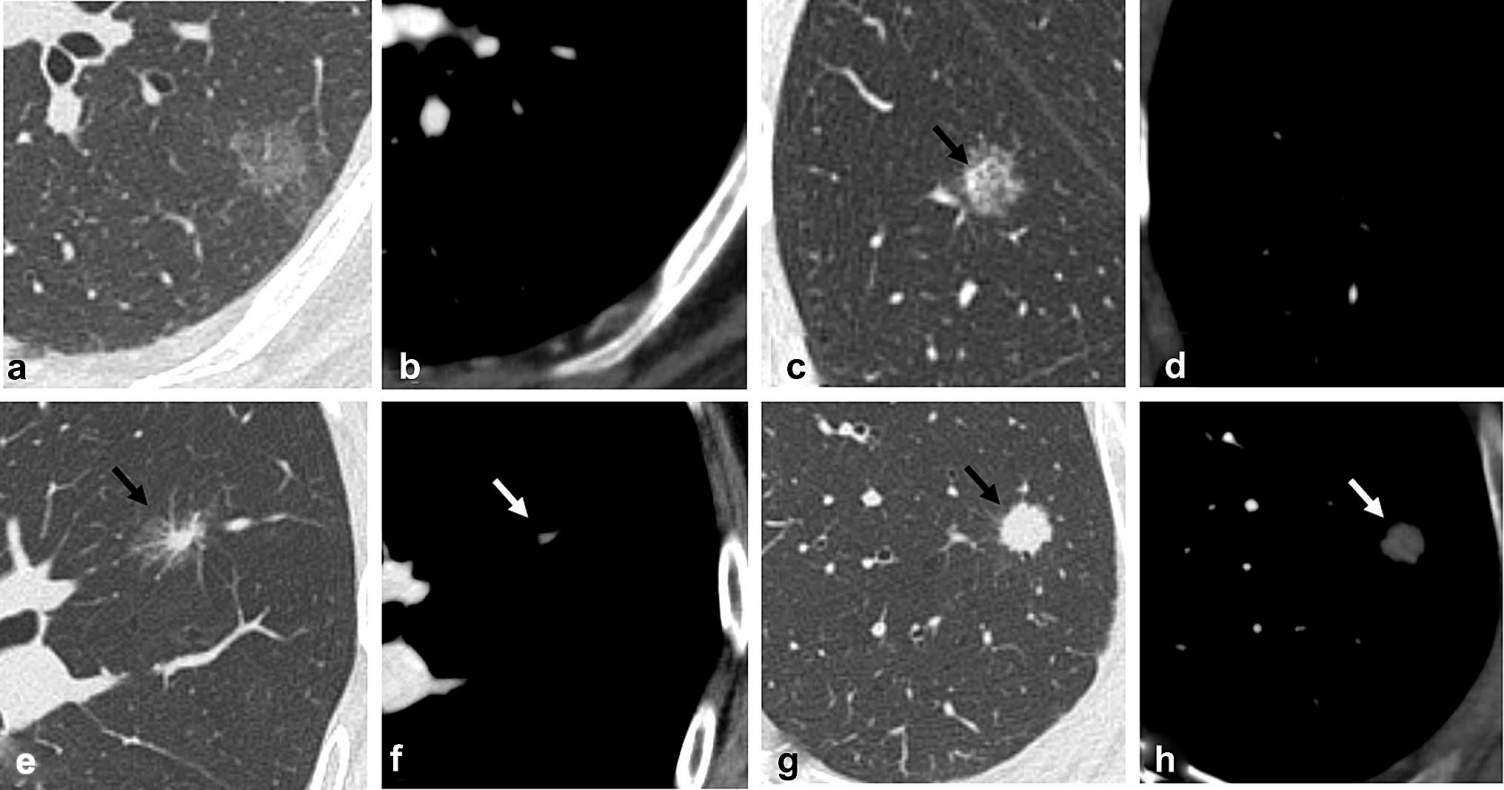
Classification of pulmonary nodules **a** Pure ground-glass nodules (GGNs) consisted of homogeneous opacities when viewed using the lung window. **b** No solid component was observed when they were viewed with the mediastinal window. **c** and **d** Heterogeneous GGNs demonstrated a solid component (black arrow) when viewed with the lung window, but not the mediastinal window. **e** and **f** Part-solid nodules were defined as GGNs that exhibited a solid component on both the lung (black arrow) and mediastinal (white arrow) windows. Solid nodules exhibited a solid component without any ground-glass opacity on both the lung (black arrow) and mediastinal (white arrow) windows

The main primary lung cancer (histologically proven pulmonary Ad) was visually classified into four groups based on TSCT (Fig. 1): pure GGNs, heterogeneous GGNs, part-solid nodules, and solid nodules. In cases in which two or more Ad-containing nodules were seen in the lung on preoperative TSCT, we defined the nodule that contained the largest (in proportional terms) solid component as the main primary pulmonary Ad, which was resected in all cases. The mean time between the surgery and preoperative CT was 14.3 ± 14.0 (1–57) days.

The surgically resected pulmonary Ads were evaluated pathologically, and the histological sub-classification of Ad was determined according to a classification of pulmonary Ad introduced by the International Association for the Study of Lung Cancer, the American Thoracic Society, and the European Respiratory Society [13]. The pathological cancer stage was assessed based on the TNM classification of the International Union Against Cancer [14].

Information was obtained for all survivors, either during hospital visits or by telephone interviews with the patient or a relative. The mean follow-up time was 7.07 ± 2.68 years. The last update of data took place in February 2021. Multiple primary lung tumors were diagnosed based on the criteria for diagnosing multiple primary carcinomas of the lung reported by Martini and Melamed et al. [15]. In 1975. Tumors were defined as ‘synchronous’ when they were detected or resected simultaneously and as ‘metachronous’ when the second tumor was found later.

### CT scanning

The CT scans were obtained using a helical technique with a 16-detector-row CT scanner (Toshiba Aquilion 16) or a 64-detector-row CT scanner (Toshiba Aquilion 64 and SOMATOM Definition, Siemens). Helical CT scans were obtained from the lung apex to the lung base. For all multi-detector-row CT scans, a section thickness of 1 mm, a helical pitch of 0.9, a reconstruction interval of 1 mm, a reconstruction with a lung kernel (FC51) and a mediastinum kernel (FC13), a tube potential of 120 kVp, and a tube current of 150 mA were used for scanning and image reconstruction. In 258 (80.4%) out of 321 cases, contrast-enhanced CT scans were performed using contrast media (Iopamidol 370; Bayer Yakuhin, Osaka) at 4.0 mL/s (total volume, 1 mL/kg) with a 20-mL saline chaser at 4.0 mL/s.

### Interpretation of the CT scans

The CT scans were evaluated independently by two chest radiologists (10 and 16 years of experience in reading chest CT scans). Pulmonary nodules on TSCT were visually classified into four groups: pure GGNs, heterogeneous GGNs, part-solid nodules, and solid nodules (Fig. 1). A pure GGN was defined as a nodule that showed a homogeneous hazy area of increased opacity when viewed using the lung window. A heterogeneous GGN was defined as a nodule that showed both a region of ground-glass opacity (GGO) and a solid portion when viewed with the lung window, but did not exhibit a solid component when viewed with the mediastinal window. A part-solid nodule was defined as a nodule that exhibited both a region of GGO and a solid portion on both the lung and mediastinal windows. A solid nodule was defined as a nodule that showed a solid component without any GGO on both the lung and mediastinal windows.

The images were viewed with the following lung window settings: The lung windows were set at a window width of 1600 Hounsfield units (HU) and a window level of − 600 HU. The mediastinal windows used for the evaluation of the presence of solid components were set at a window width of 300 HU and a window level of 25 HU. If there was a disagreement regarding the classification of a nodule between the two observers, the final decision was made by an adjudicator (34 years of experience in reading chest CT scans).

Moreover, the two chest radiologists independently measured the maximum total and solid part diameter of the heterogeneous GGNs and part-solid nodules on axial images at the lung window settings. The average value of them measured by the two readers was used for the analysis in this study.

### Standard institutional protocols before and after operation

Contrast-enhanced MRI to search for brain metastases and chest and abdominal CT to search for whole body metastases are performed before operation. After the operation, chest CT is performed to search for the recurrence once every six months for the first two years. After that, if there are no signs of recurrence, it is performed once a year. Brain MRI and bone scintigraphy are not be performed regularly, but should be performed as needed, such as when symptoms appear.

### Statistical analysis

SPSS for Windows, version 24 (SPSS Inc., Chicago, IL, USA), was used to conduct all statistical analyses. Continuous variables are presented as the mean ± standard deviation, and categorical data are presented as counts and proportions. The patients’ characteristics and the size and the pathological findings of the pulmonary Ad were compared between the heterogeneous GGNs and part-solid nodules using the Kruskal–Wallis test, Chi-square test, or Cochran–Armitage test with Bonferroni’s correction, as appropriate. Disease-free survival (DFS) was defined as the time from surgery to documented clinical recurrence, death, or the last follow-up. Overall survival (OS) was defined as the time from surgery to death or the last follow-up. Prognosis was analyzed using the Kaplan–Meier method and differences were determined using the log-rank test. *P* values of < 0.05 were considered to indicate significance.

## Results

### TSCT-based classification of pulmonary Ad according to postoperative stage

According to TSCT, the stage IA pulmonary Ad of the 321 patients included 70 solid nodules, 181 part-solid nodules, 61 heterogeneous GGNs, and 9 pure GGNs. The inter-observer agreement for the classification was good (*k* = 0.78, *p* < 0.0001).

### Characteristics of the patients with heterogeneous GGN and part-solid nodule

Table 1 shows the clinical characteristics of the 242 patients with stage IA heterogeneous GGNs or part-solid nodules. There were no significant differences between the two groups in age, sex, smoking habits, the frequency of malignant disease complications and a history of lung cancer surgery.

### Size of heterogeneous GGNs and part-solid nodules

The maximum total diameter of the 61 heterogeneous GGNs ranged from 7 to 26.5 mm (mean 15.9 mm), whereas that of the 181 part-solid nodules ranged from 9 to 47.5 mm (mean 19.8 mm). There were significant differences between them (*p* < 0.001). The maximum solid part diameter of the 61 heterogeneous GGNs ranged from 2 to 9.5 mm (mean 4.6 mm), whereas that of the 181 part-solid nodules ranged from 2.5 to 38.5 mm (mean 11.9 mm). There were significant differences between them (*p* < 0.001) (Table 2). Inter-observer agreement regarding the size was excellent (intra-class correlation coefficient (ICC) values, 0.934 [95% CI, 0.922–0.945]).

**Table 2:** Size of heterogeneous GGNs and part-solid nodules (*n* = 242)

Variables	Heterogeneous GGNs (*n* = 61)	Part-solid nodules (*n* = 181)	*P* value
Total size (mm)	15.9 ± 4.8	19.8 ± 6.1	< 0.001
Solid size (mm)	4.6 ± 1.5	11.9 ± 5.9	< 0.001

Data are expressed as n or mean ± standard deviation (range) values. *GGN* ground-glass nodule

### Pathological characteristics of resected pulmonary Ads with heterogeneous GGN and part-solid nodule

Table 3 shows the pathological characteristics of 242 resected pulmonary Ads with heterogeneous GGN and part-solid nodule, which were classified according the system developed by the International Association for the Study of Lung Cancer, the American Thoracic Society, and the European Respiratory Society [13]. 110 nodules evaluated in a previous classification system were also reviewed with a pathologist and included in this study. There were 181 part-solid nodules, which were classified into 116 invasive Ads (64.1%), 54 MIAs (29.8%), and 11 Ad in situ (AIS) lesions (6.1%). There were 61 heterogeneous GGNs, which were classified into 18 invasive adenocarcinomas (29.5%), 25 MIAs (41.0%), and 18 AIS lesions (29.5%). In terms of the pathological classification, there were significant differences between the part-solid nodules and heterogeneous GGNs (*p* < 0.001).

**Table 3:** Pathological characteristics of resected pulmonary Ad with heterogeneous GGN and part-solid nodule (*n* = 242)

Variables	Heterogeneous GGNs (*n* = 61)	Part-solid nodules (*n* = 181)	*P* value
Invasive adenocarcinoma (*n*)	18 (29.5%)	116 (64.1%)	
Minimally invasive adenocarcinoma (*n*)	25 (41.0%)	54 (29.8%)	< 0.001
Adenocarcinoma in situ (*n*)	18 (29.5%)	11 (6.1%)	

*Ad* adenocarcinoma, *GGN* ground-glass nodule

### DFS and OS

**Fig. 2:**
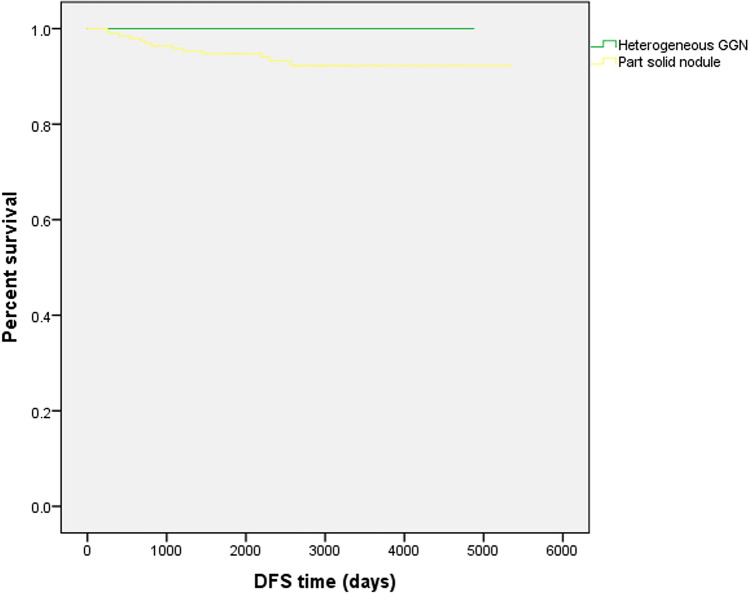
Kaplan–Meier survival curves of DFS The curves were created using data for 61 patients with heterogeneous GGNs and 181 patients with part-solid nodules. The green and yellow curves indicate the percent survival of the patients with heterogeneous GGNs and part-solid nodules, respectively. Heterogeneous GGNs were associated with significantly longer DFS than part-solid nodules

There were no cases of recurrence from pulmonary Ad in the heterogeneous GGN group. Recurrence was observed in 12 (6.6%) of 181 patients in the part-solid nodule group. The recurrence involved intrapulmonary metastasis (*n* = 6), local recurrence (*n* = 2), lymph node metastasis (*n* = 1), brain metastasis (*n* = 2), and adrenal metastasis (*n* = 1). Figure 2 shows the DFS curves of the two groups. Kaplan–Meier analysis demonstrated that heterogeneous GGNs were associated with significantly longer DFS than part-solid nodules (*p* = 0.042).

**Fig. 3:**
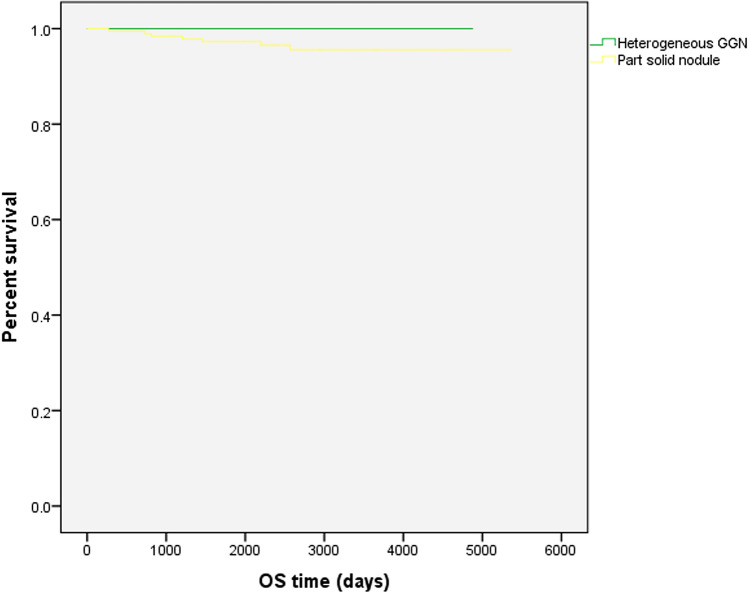
Kaplan–Meier survival curves of OS The curves were created using data for 61 patients with heterogeneous GGNs and 181 patients with part-solid nodules. The green and yellow curves indicate the percent survival of the patients heterogeneous GGNs and part-solid nodules, respectively

Death from the primary disease occurred only in 6 (3.3%) of 181 patients in the part-solid nodule group. Figure 3 shows the OS curves of the two groups. There was no significant difference in OS between heterogeneous GGNs and part-solid nodules (*p* = 0.134).

## Discussion

Our study aimed to clarify the characteristics of heterogeneous GGNs in stage IA pulmonary Ad. There were no cases of the recurrence of or death due to pulmonary Ad in the heterogeneous GGN group after surgery. Heterogeneous GGNs were associated with significantly longer DFS than part-solid nodules. Pathologically, there were significant differences between them.

In general, pure solid nodules of pulmonary Ad that do not exhibit a GGO component on TSCT have been found to exhibit more malignant behavior and have a worse prognosis than part-solid pulmonary Ads [16, 17]. The presence of GGO on TSCT always suggests a favorable surgical outcome in cases of early-stage pulmonary Ad [18, 19]. According to the guidelines developed by the Fleischner Society in 2017, SSNs were classified into pure GGNs that only possess a GGO component and part-solid nodules with both GGO and solid components, according to TSCT. According to this classification, both the heterogeneous GGNs and part-solid nodules in the present study should be categorized as part-solid nodules. In the current study, a heterogeneous GGN was defined as a nodule that showed both a region of GGO and a solid portion when viewed with the lung window, but did not exhibit a solid component when viewed with the mediastinal window.

Few studies have examined heterogeneous GGNs. In 2016, Kakinuma et al. [12] showed that the growth rates of pure GGNs, heterogeneous GGNs, and part-solid nodules differed. Among 1046 pure GGNs, 13 (1.2% [13 of 1046]) developed into heterogeneous GGNs and 56 (5.4% [56 of 1046]) developed into part-solid nodules. Among 81 heterogeneous GGNs, 16 (19.8% [16 of 81]) developed into part-solid nodules. On average, it took the 56 pure GGNs and 16 heterogeneous GGNs 3.8 ± 2.0 years and 2.1 ± 2.3 years, respectively, to develop into a part-solid nodule (*p* = 0.0004). Moreover, the 2-year and 5-year estimated probabilities of nodule growth of ≥ 2 mm were 2 and 14% for pure GGNs, 12 and 24% for heterogeneous GGNs, and 17 and 48% for part-solid nodules, respectively. The 2-year and 5-year estimated probabilities of solid component growth of ≥ 2 mm were 0 and 2% for pure GGNs, 0 and 5% for heterogeneous GGNs, and 9 and 22% for part-solid nodules, respectively. These results suggested that the characteristics of pure GGNs, heterogeneous GGNs, and part-solid nodules are different. Pathologically, the 7 resected heterogeneous GGNs examined in the latter study consisted of 5 MIAs and 2 AIS. In our study, heterogeneous GGNs and pure GGNs were only found in patients with postoperative stage IA pulmonary Ad. The maximum total and solid part size of part-solid nodules were larger than those of heterogeneous GGNs (*p* < 0.001, *p* < 0.001). Pathologically, the 32 resected heterogeneous GGNs consisted of 9 invasive Ads, 15 MIAs, and 8 AIS. There were significant differences between the pathological diagnoses of part-solid nodules and heterogeneous GGNs (*p* < 0.001).

To the best of our knowledge, there is only one previous report about the DFS and OS of patients with resected heterogeneous GGNs. Lai et al. [20] reported that the 5-year DFS in patients with part-solid nodule was significantly worse than that of patients with heterogeneous GGN or pure GGN (91.9% vs 100% vs 100%, *P* < 0.001). No patients with heterogeneous GGN or pure GGN experienced relapse or death due to lung cancer during the follow-up period. In our study, none of the patients in whom heterogeneous GGNs were resected experienced recurrence. On the other hand, recurrence was observed in 12 (6.6%) of 181 patients in the part-solid nodule group. Heterogeneous GGNs were associated with significantly longer DFS than part-solid nodules. As a result, we speculate that heterogeneous GGNs have less malignant potential than part-solid nodules. On the other hand, the OS of the heterogeneous GGN group tended to be better than that of the part-solid nodule group, but the difference was not significant. Death from the primary disease occurred in 6 (3.3%) of 181 patients in the part-solid nodule group, which was much lower than the rate of recurrence. One plausible reason for this is that the recent development of drug therapies, including immune-checkpoint inhibitors, has improved the survival of patients with recurrent lung cancer [21].

In the guidelines published by the Fleischner Society in 2017, both heterogeneous GGNs and part-solid nodules were categorized as part-solid nodules. However, the current study suggested that heterogeneous GGNs and part-solid nodules have different characteristics, and part-solid nodules have much greater malignant potential than heterogeneous GGNs. On the basis of these findings, more careful follow-up should be considered for part-solid nodules than for heterogeneous GGNs.

### Limitations

This study had several limitations. First, it was conducted retrospectively. Therefore, the results cannot be generalized to the entire population because this study involved a specially selected subset of the population. Second, the differentiation between heterogeneous GGNs and part-solid nodules was performed based on visual assessments, and no quantitative evaluations based on the CT values of solid components were carried out. This might affect comparisons with results from other institutions. Despite these limitations, the results of this study are clinically useful and will aid the decision-making of clinicians and radiologists who encounter GGNs in patients with pulmonary Ad.

## Conclusion

Heterogeneous GGNs are associated with longer DFS than part-solid nodules. Pathologically, there are significant differences between part-solid nodules and heterogeneous GGNs. Heterogeneous GGNs may have less malignant potential than part-solid nodules.
